# Effect of TCM rehabilitation program on activities of daily living in patients with post-stroke limb spasticity: An observational study

**DOI:** 10.1097/MD.0000000000036079

**Published:** 2023-11-24

**Authors:** Chuanxi Zhu, Long Qiu, Weichen Sun, Chong Yang, Deyu Cong, Yufeng Wang, Guangcheng Ji

**Affiliations:** a Department of Rehabilitation Medicine, Changchun University of Chinese Medicine, Changchun, China; b Department of Acupuncture and Tuina, Changchun University of Chinese Medicine, Northeast Asia Research Institute of Traditional Chinese Medicine, Changchun, China; c Changchun University of Chinese Medicine, Changchun, China; d Department of Rehabilitation, The Third Affiliated Hospital of Changchun University of Chinese Medicine, Changchun, China.

**Keywords:** activities of daily living, quality of life, spasm, stroke, traditional Chinese medicine (TCM) rehabilitation program

## Abstract

**Background:**

Stroke is a neurological disease with many common complications that reduce the activities of daily living and the quality of life of patients. Traditional Chinese medicine (TCM) rehabilitation techniques, scalp acupuncture, and TCM can relieve spasticity symptoms and recovery from physical obstacles is significant.

**Methods:**

Three hundred twenty-one patients with post-stroke limb spasticity were randomly divided into trial and control groups, with 159 and 162 patients in the trial and control groups, respectively. The control group received basic treatment combined with modern rehabilitation techniques, whereas the trial group received basic treatment combined with TCM, Tuina, and scalp acupuncture with kinesiotherapy. The treatment course in both groups was 4 weeks. The Modified Ashworth Scale, magnetic resonance imaging, and Stroke Specific Quality of Life Scale were used to evaluate limb spasticity, activities of daily living, and quality of life, respectively. PASW 18.0 was used for statistical analysis.

**Results:**

With a longer treatment period, the improvement in limb spasticity was greater in the trial group than in the control group (*P* < .05). Similarly, improvements in activities of daily living and quality of life were better in the trial group than in the control group (*P* < .05).

**Conclusion:**

The TCM rehabilitation program using Tongjing Tiaoxing combined with scalp acupuncture and kinesiotherapy can effectively treat spasticity symptoms in stroke patients and improve their activities of daily living and quality of life.

## 1. Introduction

A survey published by a study group on the epidemiology of cerebral infarction in China showed that stroke ranked first among the causes of disability and death. Strokes can be classified into 2 main categories according to the pathogenesis and course of the disease: hemorrhagic and ischemic.^[1,2]^ Of the many stroke complications, limb spasms are the most common with 4% (14 weeks after stroke) to 42.6% (1–3 months after stroke) of patients affected by varying degrees of spasms.^[3–5]^ Spasticity is one of the main factors that restrict body movement. Patients with limb spasms often present with abnormal movement patterns due to elevated muscle tension in the dominant muscle groups that are observed during elbow flexion in the upper limbs and knee extension in the lower limbs.^[6,7]^ This not only affects the rehabilitation efficacy of patients but also leads to a longer treatment cycle and reduces the patients’ ability to perform activities of daily living and their quality of life, resulting in a huge medical burden on families and society.^[8–10]^ Treatment with conventional Traditional Chinese Medicine (TCM) rehabilitation techniques, scalp acupuncture, and classic TCM formulas can alleviate spastic symptoms to a certain extent, which is significant in promoting recovery from limb movement disorders. However, problems such as overlap in many effective interventions, missing theoretical guidance and clinical basis for these interventions, lack of a systematic TCM rehabilitation program, and inability to implement standard treatments continue to exist. Therefore, there is a pressing need to develop a scientific, standardized, and efficient comprehensive TCM rehabilitation program. This study aimed to determine the effectiveness of a TCM rehabilitation program on the ability to perform activities of daily living and quality of life in patients with post-stroke limb spasticity at different times to identify a better treatment program for post-stroke limb spasticity.

## 2. Subjects and methods

### 2.1. Study subjects

**Figure 1.**
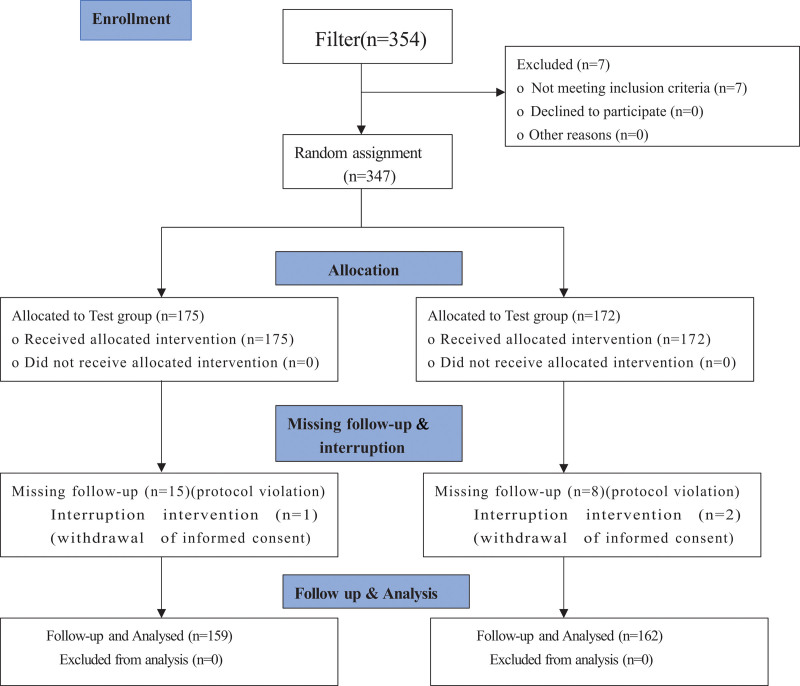
Trial flow chart.

From December 2019 to December 2022, 77 patients from the Second Affiliated Hospital of Heilongjiang University of Chinese Medicine, 36 patients from Huashan Hospital of Fudan University, and 208 patients from Changchun University of Chinese Medicine, a total of 321 cases who met the inclusion criteria were enrolled in this study (Fig. 1). All cases that met the inclusion criteria were randomly assigned to either the trial or the control group. There were 120 males and 39 females, 159 cases in total, in the trial group, with an average age of (56.3 ± 10.41) years old, average body temperature (°C) of (36.43 ± 0.18) °C, average heart rate (beats/minute) of (77.44 ± 12.04) beats/minute and average respiratory rate (breaths/minute) of (18.08 ± 1.01) breaths/minute. There were 131 males and 31 females, 162 cases in total, in the control group with an average age of (56.83 ± 9.95) years old, average body temperature (°C) of (36.42 ± 0.20) °C, average heart rate (beats per minute) of (78.64 ± 11.97) beats per minute, and average respiratory rate (breaths/minute) of (18.07 ± 0.70) breaths/minute. There were no statistically significant differences in sex, age, body temperature, heart rate, or respiratory rate between the 2 groups of patients (*P* > .05).

#### 2.1.1. Diagnostic criteria.

The diagnostic criteria according to Western medicine were based on the diagnostic criteria for cerebral infarction and cerebral hemorrhage in the Chinese Guidelines for Diagnosis and Treatment of Acute Ischemic Stroke 2018^[11]^ and the Chinese Guidelines for Diagnosis and Treatment of Subarachnoid Hemorrhage 2019.^[12]^ The diagnostic criteria were based on the Chinese medicine guidelines for the diagnosis and treatment of common internal diseases.^[13]^ All cases were confirmed by CT or magnetic resonance imaging.

#### 2.1.2. Inclusion criteria.

(1) Diagnosis was in line with the diagnostic criteria in both Western medicine and traditional Chinese medicine for strokes; (2) an increase in limb muscle tension with a score of 1 to 2 on the Modified Ashworth Scale; (3) duration of the disease between 2 weeks and 6 months; (4) Aged 35–70 years old; (5) those with a clear mind and stable vital signs; and (6) patient or the patient’s family had signed the informed consent form.

#### 2.1.3. Exclusion criteria.

(1) Limb spasms not due to stroke; (2) complications of severe primary diseases involving organs such as the liver, kidneys, and hematopoietic and endocrine systems; (3) symptoms such as severe visual, auditory, cognitive impairment, and mental illness, and inability to cooperate with examination and treatment; (4) pregnancy and lactation; and (5) unstable vital signs.

### 2.2. Treatment methods

Standard basic treatment measures with reference to the China Guidelines for the Prevention and Treatment of Cerebrovascular Diseases developed by the Department of Disease Control of the Ministry of Health and the Neurological Society of the Chinese Medical Association in 2005 were adopted for both trial and control groups. Individualized treatment was used to control and stabilize blood pressure to ≤135/85 mm Hg or within the normal range. Appropriate hypoglycemic drugs were selected to control and maintain normal blood glucose levels. Similarly, appropriate lipid-lowering drugs were chosen based on triglyceride and cholesterol levels. Symptomatic treatment, prevention and treatment of complications were assessed.

#### 2.2.1. Control group.

(1) In addition to basic treatment, kinesiotherapy training according to the rehabilitation program in the 2017 Rehabilitation Diagnosis and Treatment Program and Clinical Pathway by the National Administration of TCM was performed in the control group during the spasticity period. The specific operations were as follows: ① muscle spasm control: placement of good limbs; methods such as the reflex-inhibiting pattern in the Bobath technique, tension influencing posture (TIP), key pointers on control; and Rood’s approach to sensory stimulation, suppressed spasms using corresponding sensory stimuli. ② Facilitating the emergence of isolated movements: Employed training such as neural facilitation techniques and motor relearning to further facilitate isolated movements of the affected limb. ③ Therapeutic training: sitting and standing balance training, as well as training to walk and climb stairs; The intervention time was 30 minutes per session, once per day, with 5 days of treatment per week, with a treatment course of 4 weeks.

#### 2.2.2. Trial group.

(1) The enrolled patients were orally administered TCM tablets designed to nourish the brain for recovery. The Specific prescriptions were as follows: 10 g red peony, 6 g chuanxiong, 10 g pollen typhae, 10 g pueraria, 10 g raw sophora flower, 10 g pheretima, 6 g safflower, 15 g siegesbeckiae herb, and 9 g pseudo-ginseng. One portion per dose was divided into 2 doses by either oral (with water) or nasal feeding, and a treatment course of 4 weeks. (2) Tuina treatment uses massage techniques such as rolling, kneading, tapping, holding, and stretching. The governor vessel meridian was chosen to improve circulation in the body, and the yin and yang meridians of the upper and lower limbs were chosen to improve circulation in the affected limb, as well as the following acupoints on the affected side: Quchi, Hegu, Weizhong, Yanglingquan, and Taichong. Intervention timing and duration: Each treatment lasted about 30 minutes, and was administered once a day, 5 times a week for 4 weeks. (3) Acupuncture kinesiotherapy was performed at acupoints at the top and anterior of the head. The skin around the acupoints was first disinfected with a 75% alcohol cotton ball. Then, using either the hold-and-insert needling method or the press-and-insert needling method, a 40 mm × 0.35 mm needle was used to quickly puncture the acupoints at an angle of 30° and slowly inserted until the needle was horizontal to the underside of the galea, and had penetrated 1 to 1.5 inches. Subsequently, the KWD-8081 electro acupuncture simulator was used to connect the acupoints of Baihui and Qianding (both sides) with a dense waveform setting in which the patient was able to withstand 30 minutes of electroacupuncture treatment, after which the needles were left on the acupoints for 6 hours. With the needles on the acupoints, kinesiotherapy training during the period of spasticity was performed for 30 minutes with reference to the 2017 Rehabilitation Diagnosis and Treatment Program and Clinical Pathway by the National Administration of TCM. The intervention timing and duration were a total of 6 hours of treatment per session, once per day, 5 days per week for a treatment course of 4 weeks.

### 2.3. Observation indicators and evaluation timeline

(1) Spasticity was assessed using the Modified Ashworth Scale, (2) the ability to perform activities of daily living was assessed using the Modified Bathel Index, and (3) the Stroke Specific Quality of Life Scale (SS-QOL) was used to assess the quality of life. (4) Evaluation timeline: ① Clinical evaluation timeline: before intervention (V1), 2 weeks after intervention (V2), and 4 weeks after intervention (V3); ② Follow up evaluation timeline: 3 months (V4) and 6 months (V5) after the end of intervention.

### 2.4. Statistical analysis

PASW 18.0 (IBM SPSS Inc., Armonk, NY) was used for statistical analysis, and the chi-squared test was used for count data. The measurement data are expressed as $\bar{x}$ ± s, and *t* tests were applied. Differences were considered statistically significant at *P* < .05.

## 3. Results

### 3.1. Comparison of the modified Ashworth scores for elbow extension before and after intervention

**Figure 2.**
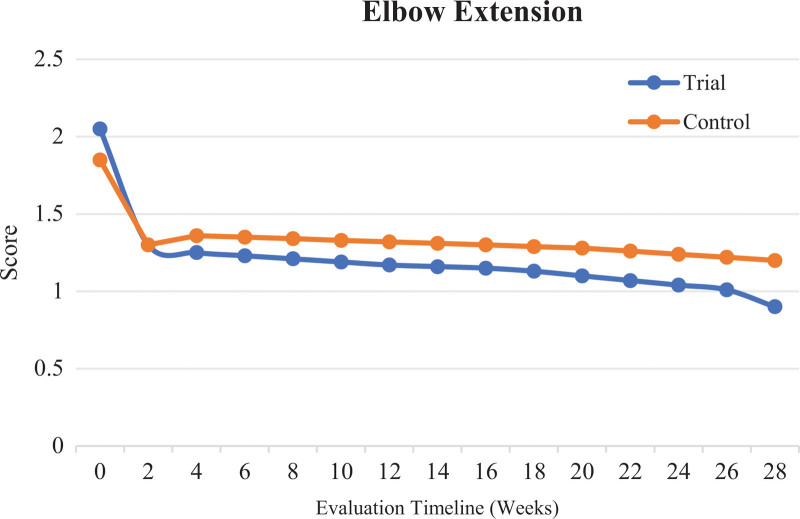
Comparison of Modified Ashworth scores for elbow extension in the trial and control groups before and after the intervention.

The results in Table 1 show statistically significant differences (*P* < .05) in changes to the Modified Ashworth scores for elbow extension between the groups at 6 months (V5) after the end of intervention, the period from before intervention to after 4 weeks of intervention (V3 − V1), and the period from before intervention to 6 months after the end of intervention (V5 − V1) (*P* < .05). There were no statistically significant differences in the other indicators between the groups. This suggests that with a longer treatment duration, the improvement in elbow extension spasm was better in the trial group than in the control group, as shown in Figure 2.

**Table 1:** Comparison of the Modified Ashworth scores for elbow extension before and after intervention $\left(\bar{x}\pm s\right)$.

Group	N	V1	V2	V3	V4	V5	V3 − V1	V5 − V1
Trial	159	2.050 ± 1.011	1.82 ± 0.958	1.25 ± 0.871	1.11 ± 0.857	0.9 ± 0.773	−0.799 ± 0.673	−1.151 ± 0.789
Control	162	1.944 ± 1.011	1.8 ± 1.052	1.33 ± 0.89	1.25 ± 0.849	1.11 ± 0.863	−0.617 ± 0.622	−0.833 ± 0.75
*t*		0.938	0.246	−0.769	−1.404	−2.316	−2.51	−3.697
*P*		0.349	0.806	0.443	0.161	0.021	0.013	<0.001

### 3.2. Comparison of the modified Ashworth scores for knee flexion before and after intervention

**Figure 3.**
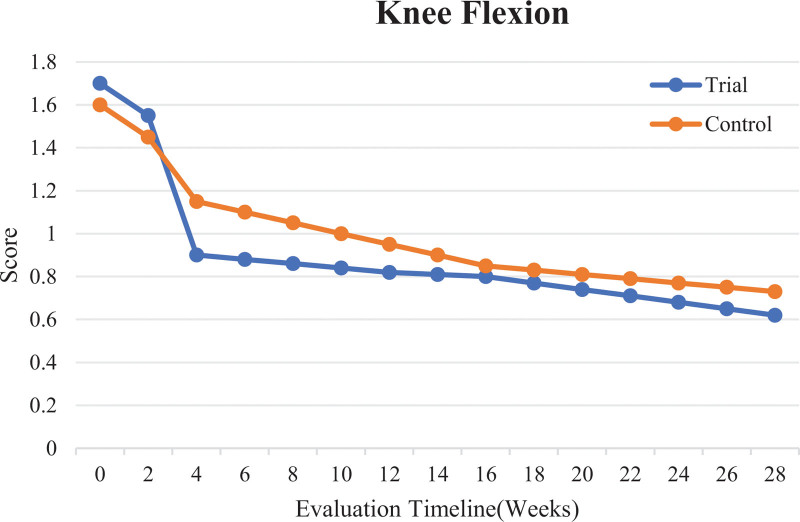
Comparison of Modified Ashworth scores for knee flexion in the trial and control groups before and after intervention.

The results in Table 2 show statistically significant differences (*P* < .05) in changes to the Modified Ashworth scores for knee flexion between the 2 groups in the period from before intervention to after 4 weeks of intervention (V3 − V1). However, there were no statistically significant differences in other indicators between the groups. This suggests that, with a longer treatment duration, the trial group will show more improvement in knee joint spasms than the control group, as shown in Figure 3.

**Table 2:** Comparison of the Modified Ashworth scores for knee flexion before and after intervention $\left(\bar{x}\pm s\right)$.

Group	N	V1	V2	V3	V4	V5	V3 − V1	V5 − V1
Trial	159	1.686 ± 0.982	1.55 ± 0.979	0.94 ± 0.765	0.88 ± 0.758	0.65 ± 0.711	−0.742 ± 0.668	−1.031 ± 0.853
Control	162	1.611 ± 1.053	1.46 ± 0.991	1.12 ± 0.908	0.9 ± 0.896	0.72 ± 0.851	−0.494 ± 0.603	−0.889 ± 0.78
*t*		0.655	0.822	−1.857	−0.157	−0.779	−3.413	−1.563
*P*		0.513	0.412	0.064	0.875	0.437	0.001	0.119

### 3.3. Comparison of the modified Barthel Index before and after intervention

**Figure 4.**
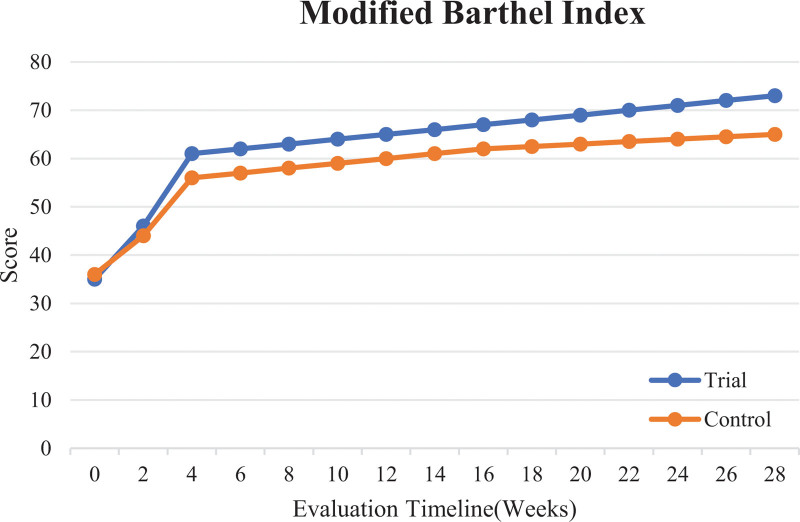
Comparison of the Modified Barthel Index in the trial and control groups before and after intervention.

The results in Table 3 show statistically significant differences (*P* < .05) in changes in the Modified Barthel Index between the 2 groups after 4 weeks of intervention (V3), 3 months (V4) and 6 months (V5) after the end of intervention, the period from before intervention to after 4 weeks of intervention (V3 − V1), and the period from before intervention to 6 months after the end of intervention (V5 − V1). There were no statistically significant differences in the other indicators between the groups. This suggests that with a longer treatment duration, along with improvements in spasticity, the trial group showed greater improvement in the ability to perform activities of daily living than the control group, as shown in Figure 4.

**Table 3:** Comparison of the Modified Barthel Index before and after intervention $\left(\bar{x}\pm s\right)$.

Group	N	V1	V2	V3	V4	V5	V3 − V1	V5 − V1
Trial	159	34.97 ± 19.31	45.95 ± 17.28	60.59 ± 15.22	66.05 ± 15.92	69.57 ± 17.58	25.62 ± 14.82	34.59 ± 18.48
Control	162	36.15 ± 19.71	44.01 ± 17.74	56.80 ± 14.61	61.71 ± 14.80	64.43 ± 16.40	20.65 ± 13.09	28.27 ± 18.04
*t*		−0.541	0.994	2.275	2.530	2.709	3.185	3.1
*P*		0.589	0.321	0.024	0.012	0.007	0.002	0.002

### 3.4. Comparison of the overall scores in the SS-QOL before and after intervention

**Figure 5.**
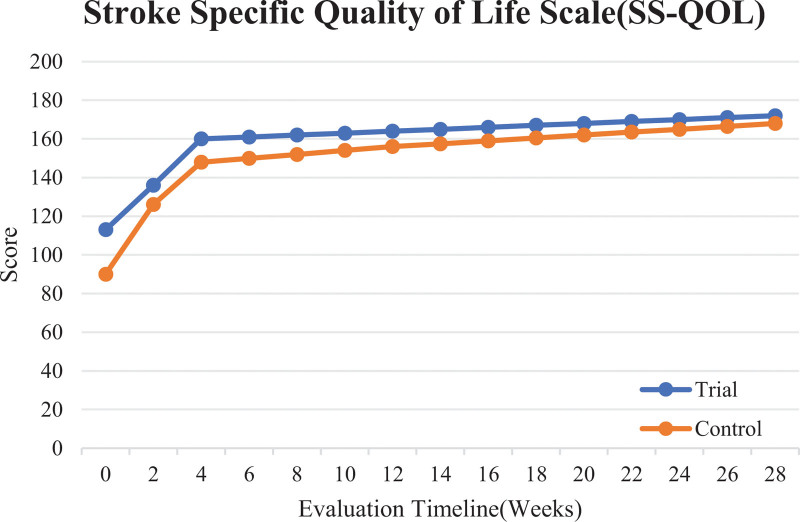
Comparison of the overall scores of the SS-QOL in the trial and control groups before and after intervention. SS-QOL = Stroke Specific Quality of Life Scale.

In Table 4, the results show statistically significant differences (*P* < .05) in the overall SS-QOL scores between the 2 groups after 2 weeks (V2) and 4 weeks (V3) of intervention, 3 months (V4) and 6 months (V5) after the end of intervention, the period from before intervention to after 4 weeks of intervention (V3 − V1), and the period from before intervention to 6 months after the end of intervention (V5 − V1). There were no statistically significant differences in the other indicators between the groups. This indicates that the trial group was superior to the control group in improving the quality of life of the stroke patients, as shown in Figure 5.

**Table 4:** Comparison of the overall SS-QOL scores before and after intervention $\left(\bar{x}\pm s\right)$.

Group	N	V1	V2	V3	V4	V5	V3 − V1	V5 − V1
Trial	159	113.92 ± 33.85	135.64 ± 31.51	159.69 ± 32.26	167.64 ± 35.07	175.13 ± 35.95	45.77 ± 29.05	61.21 ± 37.51
Control	162	107.81 ± 35.76	127.17 ± 33.39	148.75 ± 30.87	158.83 ± 31.68	167.14 ± 32.32	40.94 ± 29.41	59.33 ± 35.51
*t*		1.570	2.336	3.104	2.361	2.095	1.482	0.463
*P*		0.117	0.02	0.002	0.019	0.037	0.139	0.644

SS-QOL = Stroke Specific Quality of Life Scale.

## 4. Discussion

Post-stroke spasticity is a common clinical complication with most patients experiencing spasms in the affected limb. The main clinical symptoms are paralysis of the lower limb flexor and upper limb extensor muscles, and increased muscle tension.^[14]^ Various interventions are often adopted for the clinical treatment of post-stroke spasticity, including physical therapy, oral medicine, drug injections, and surgery. However, there are certain limitations to any single treatment method, and in modern medicine, there is no medicine or therapy that has proven to be fully effective for post-stroke limb spasms. Many characteristic TCM diagnoses and treatment methods, such as acupuncture, moxibustion, tuina, oral medication, and acupoint plasters, have been widely used in the treatment of post-stroke spasms and play a significant role in facilitating recovery from limb dyskinesia.^[15]^

Muscular tension is the amount of tension produced by a muscle during activity, and it is directly related to posture; body posture is determined by muscular tension. Currently, muscular tension is commonly measured in the clinic with the Modified Ashworth Scale. This scale consists of 6 grades; 0, 1, 1+, 2, 3, and 4, with grade 0 representing no increased muscle tone and grade 4 representing the highest muscle tone. Increased resistance during passive flexion of the joint represents increased extensor muscle tone, and increased resistance during passive extension of the joint represents increased flexor muscle tone.^[16]^ Many modern rehabilitation therapies are available for post-stroke spasticity. For example, Reflexive Inhibitory Pattern Therapy is a new type of rehabilitation therapy technique, based on the principle that antagonizing hyperactive muscle groups produces an inhibitory effect on spastic muscles and balances the muscle tone of the active and antagonistic muscles, thus lowering muscle tone and relieving limb spasms.^[17]^ The Bobath technique is a neurophysiological therapy that restores normal behavior and posture and motor function by reflexively inhibiting neuronal damage, decreasing muscle tone, and inhibiting abnormal motor postures.^[18]^ The Rood Technique is a treatment that uses mild stimulation or epidermal temperature stimulation in a specific area of the skin, including tapping and stimulating the surface skin, brushing the affected limb, applying ice packs, pulling on the muscles, and putting pressure on the tendons, to activate the skin receptors and promote blood flow to the affected limb, thus restoring neuromuscular function.^[19]^ TCM has a unique perspective on post-stroke spasticity and has a viable treatment program.

According to the Chi Jie Zhen Xie treatise in the Classic of the Miraculous Pivot (or Ling Shu), paralysis occurs when the body is very weak with poor circulation, and when the internal defense systems are weak, good energy flows out while bad energy remains, leading to hemiplegia. Wang Qingren from the Qing Dynasty also mentioned that paralysis in the lower half of the body was due to poor health. Therefore, the characteristic pathogenesis of spasmodic paralysis after stroke is a deficiency in yang energy, poor blood and systemic circulation, and insufficient nutrients in the meridians. In the Sheng Qi Tong Tian Treatise in the Plain Question (Su Wen), yang energy nourishes the mind and tendons. The essence of the governor vessel meridian is yang; hence, it is also considered to be the ocean of the yang meridians. Thus, regulating the governor vessel is the first step toward regaining energy in yang. Once the meridians related to the governor vessels are no longer obstructed, and the exertion of yang energy becomes normal, the limbs and muscles will be warmed and nurtured, and muscular contractions, such as spasms, can be alleviated. Therefore, acupoints on the meridian of the governor vessel were selected for the treatment. The governor vessel stores the energy of the 6 yang meridians, and the acupoints of the governor vessel meridian can be used to energize the 4 extremities and stimulate yang energy in the body.^[20]^ Therefore, it is recommended to use the Baihui acupoint on the governor vessel meridian, as the head is where all the yang meridians converge, which will stimulate the yang energy, boost the yang energy, and eliminate bad circulation. By reviewing the literature from around the world, organizing and analyzing the Tuina methods for post-strike limb spasms with the guidance of TCM academic experts such as Ren Jixue, Shi Xuemin and Wang Yongyan, the authors summarized the pathogenesis of post-stroke spasticity to be due to “damaged brain marrow, and poor circulation of the hepatic system, leading to the inability of the body to regulate the liver’s activities, perception is poor, and as the liver controls ascension, the liver blood cannot be transmitted to the muscles and knees, causing these muscles to lose nutrients, leading to spasms.”

The Tuina technique based on Tongjing Tiaoxing aims to clear blockages in the meridians, and regulate energy and blood flow so that energy and blood flow smoothly to the organs to achieve the goal of regulating the body form and treating spasms. Studies have shown that Tuina plays a significant role in the recovery of motor dysfunction in patients with stroke spasticity, promotion of muscular coordination, and strengthening of muscular control to exert a significant inhibitory effect on spasticity, enhance the motor function of limbs, and improve the ability of patients to perform activities of daily living.^[14]^

Scalp acupuncture is used to treat the communication pathways between the central nervous system and the 4 limbs, and Tuina to enrich and integrate the meridians to inject energy into the internal organs. These 2 methods combine to promote circulation in the meridians and treat the root cause of limb spasms, regulate the form of limb spasms to manage the symptoms, ultimately achieving the goal of treating both symptoms and disease, and restoring harmony in both physical form and mind. Scalp acupuncture is an important component of the acupuncture and moxibustion system, and is widely used in the clinical treatment of stroke. Similar to other acupuncture and moxibustion physiotherapies, this operation is simple at low cost, inexpensive, safe, and effective. Studies have shown that scalp acupuncture has therapeutic potential for improving motor function of the limbs and reducing the severity of spasticity.^[21]^ Scalp acupuncture with needles combined with kinesiotherapy, that is, acupuncture with rehabilitation training at the same time can improve the motor function and balance function of stroke patients significantly, promote metabolism and blood circulation in affected limbs, improve neurological function, and further enhance the patients’ ability to perform activities of daily living and their quality of life.^[22–24]^ The program to nourish the brain for recovery helps promote blood circulation, remove blockages, clear meridians, and activate organs and systems to produce a significant therapeutic effect in patients with ischemic stroke. It can reduce neurological damage, improve microcirculation, reduce blood viscosity, improve blood quality, and increase vascular elasticity.^[25]^ In this study, we gradually developed a comprehensive TCM rehabilitation program for patients with post-stroke limb spasticity by combining the Tuina technique to promote Tongjing Tiaoxing with scalp acupuncture and kinesiotherapy.

The Modified Barthel Index is specifically designed to evaluate the ability to perform activities of daily living, and is an important basis for assessing whether the patient requires life assistance. The scale includes ten items, including feeding, bathing, grooming, dressing, bowel control, bladder control, toileting, chair transfer, ambulation and stair climbing. It is used to evaluate the ability of patients to perform activities of daily living after stroke, and its reliability and validity have been validated by experts and scholars worldwide.^[26,27]^ The SS-QOL has been widely used to assess the quality of life of patients with Stroke and has been tested in clinical practice. The scale contains 49 questions involving 12 different domains, including energy, family roles, language, mobility, mood, personality, self-care, social roles, thinking, upper extremity function, vision, and work/productivity with a total of 245 points. The higher the score, the better the quality of life; the reverse is also true.^[28,29]^ The results of this study in this paper showed that with a longer treatment duration, the Modified Ashworth scores for elbow extension and knee flexion in the treatment group were better than those in the control group before and after intervention (*P* < .05), while the Modified Barthel Index and the overall score on the SS-QOL were also better in the trial group than in the control group before and after intervention (*P* < .05). This suggests that the TCM rehabilitation program to nourish the brain for recovery combined with Tuina and scalp acupuncture with kinesiotherapy can accelerate the rehabilitation progress of stroke patients, alleviate limb spasms, and thus improve their ability to perform activities of daily living and their quality of life. The improvements observed in the trial group were greater than those observed in the control group (*P* < .05).

In summary, as population aging has become an increasingly prominent issue in China today, coupled with the high incidence and severe forms of stroke, there is an urgent need to include TCM programs in the execution of the Healthy China Strategy. The comprehensive TCM rehabilitation program represented by a combination of Tuina for Tongjing Tiaoxing with scalp acupuncture and kinesiotherapy has the characteristics of “simplicity, convenience, efficacy, and cost-effectiveness,” and can effectively reduce the incidence of post-stroke spasms, increase the treatment efficacy of post-stroke spasms, improve the patients’ ability to perform activities of daily living, and improve their quality of life. Therefore, this standard treatment program should be promoted.

## Author contributions

**Data curation:** Long Qiu.

**Investigation:** Weichen Sun.

**Methodology:** Chong Yang.

**Project administration:** Deyu Cong, Yufeng Wang.

**Supervision:** Guangcheng Ji.

**Writing—review & editing:** Chuanxi Zhu.
